# 1-Benzyl-3-methyl­quinoxalin-2(1*H*)-one

**DOI:** 10.1107/S1600536810025614

**Published:** 2010-07-07

**Authors:** Youssef Ramli, Ahmed Moussaif, Hafid Zouihri, Saïd Lazar, E. M. Essassi

**Affiliations:** a Laboratoire de Chimie Hétérocyclique, Pole de Compétence PHARCHIM, Université Mohammed V-Agdal, BP 1014, Rabat, Morocco; b Laboratoires de Diffraction des Rayons X, Division UATRS, Centre National pour la Recherche Scientifique et Technique, Rabat, Morocco; c Laboratoire de Biochimie, Environnement et Agroalimentaire (URAC 36), Faculté des Sciences et Techniques Mohammedia, Université Hassan II Mohammedia-Casablana, BP 146, 20800 Mohammedia, Morocco

## Abstract

The asymmetric unit of the title compound, C_16_H_14_N_2_O, contains three independent mol­ecules. The dihedral angles between the quinoxaline and phenyl planes in the three mol­ecules are 82.58 (8), 85.66 (9) and 85.36 (9)°. The crystal packing is stabilized by C—H⋯O and C—H⋯N hydrogen bonds.

## Related literature

For the biological activity of quinoxaline derivatives, see: Kleim *et al.* (1995 ▶); Abasolo *et al.* (1987 ▶); Rodrigo *et al.* (2002 ▶); Jampilek *et al.* (2005 ▶); Sashidhara *et al.* (2009 ▶); Watkins *et al.* (2009 ▶).
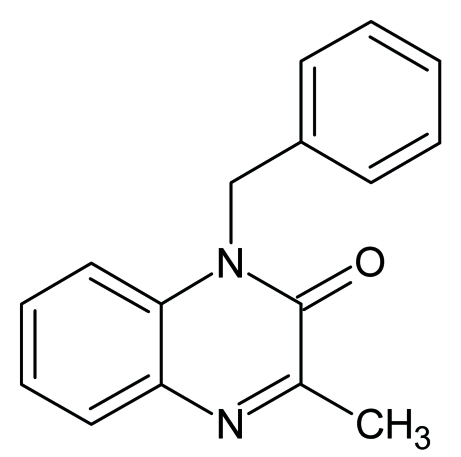


## Experimental

### 

#### Crystal data

C_16_H_14_N_2_O
*M* *_r_* = 250.29
Triclinic, 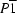

*a* = 7.4433 (2) Å
*b* = 17.5444 (5) Å
*c* = 18.0598 (7) Å
α = 118.034 (2)°
β = 100.217 (2)°
γ = 92.726 (1)°
*V* = 2025.27 (11) Å^3^
*Z* = 6
Mo *K*α radiation
μ = 0.08 mm^−1^
*T* = 296 K
0.25 × 0.21 × 0.15 mm

#### Data collection

Bruker X8 APEXII CCD area-detector diffractometer
46425 measured reflections
9750 independent reflections
6445 reflections with *I* > 2σ(*I*)
*R*_int_ = 0.028

#### Refinement

*R*[*F* ^2^ > 2σ(*F* ^2^)] = 0.050
*wR*(*F* ^2^) = 0.166
*S* = 1.09
9750 reflections
516 parameters
H-atom parameters constrained
Δρ_max_ = 0.30 e Å^−3^
Δρ_min_ = −0.24 e Å^−3^

### 

Data collection: *APEX2* (Bruker, 2005 ▶); cell refinement: *SAINT* (Bruker, 2005 ▶); data reduction: *SAINT*; program(s) used to solve structure: *SHELXS97* (Sheldrick, 2008 ▶); program(s) used to refine structure: *SHELXL97* (Sheldrick, 2008 ▶); molecular graphics: *PLATON* (Spek, 2009 ▶); software used to prepare material for publication: *publCIF* (Westrip, 2010 ▶).

## Supplementary Material

Crystal structure: contains datablocks I, global. DOI: 10.1107/S1600536810025614/pv2301sup1.cif

Structure factors: contains datablocks I. DOI: 10.1107/S1600536810025614/pv2301Isup2.hkl

Additional supplementary materials:  crystallographic information; 3D view; checkCIF report

## Figures and Tables

**Table 1:** Hydrogen-bond geometry (Å, °)

*D*—H⋯*A*	*D*—H	H⋯*A*	*D*⋯*A*	*D*—H⋯*A*
C125—H125⋯N12^i^	0.93	2.62	3.423 (2)	145
C321—H321⋯O1^ii^	0.93	2.56	3.320 (3)	140
C325—H325⋯N32^iii^	0.93	2.51	3.380 (3)	155

Symmetry codes: (i) 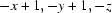; (ii) 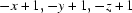; (iii) 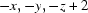.

**Fig. 1.**
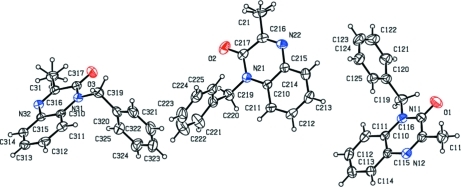
Molecular structure of the title compound with the atom-labelling scheme. Displacement ellipsoids are drawn at the 50% probability level. H atoms are represented as small spheres of arbitrary radii.

**Fig. 2.**
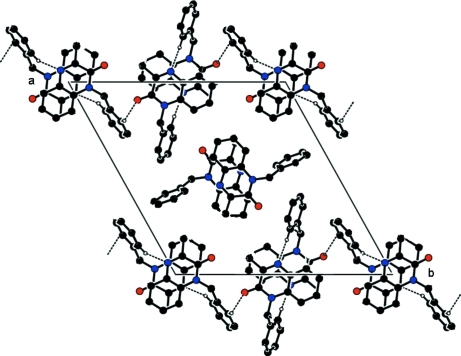
Crystal packing of the title compound viewed down the c- axis; H-atoms not involved in H-bonds have been omitted for clarity.

## supplementary crystallographic information

### Comment

Quinoxaline derivatives are an important class of nitrogen containing heterocycles in medicinal chemistry. They exhibit antimicrobial (Kleim *et al.*, 1995), antitumor (Abasolo *et al.*, 1987), and antituberculous activity (Rodrigo *et al.*, 2002]. They also exhibit antifungal, herbicidal, antidyslipidemic and antioxidative activities (Jampilek *et al.*, 2005; Sashidhara *et al.*, 2009; Watkins *et al.*, 2009). In this paper, the synthesis and crystal structure of the title compound is presented.

The asymmetric unit of the title compound contains three independent molecules (Fig. 1). The dihedral angles between the quinoxaline and phenyl planes in the three molecules are 82.58 (8), 85.66 (9) and 85.36 (9)°. The crystal structure is devoid of classical hydrogen bonds. However, the crystal packing is stabilized by C—H···O and C—H···N hydrogen bonds (Tab. 1 & Fig. 2).

### Experimental

To a solution of 3-methylquinoxali-2(1*H*)-one (1 g) in 20 ml of dimethylformamide were added benzylchloride (0.72 ml), K_2_CO_3_ (0.90 g) and catalytic amont of tetrabutylammonium bromide. The mixture was stirred at room temperature for 24 h. The solvent was removed under reduce pressure and the residue was crystallized in ethanol to afford the crystals of the title compound which were suitable for X-ray analysis.

### Refinement

All H atoms were fixed geometrically and treated as riding with C—H = 0.96 Å (methyl), 0.98Å methine or 0.93 Å (aromatic) with *U*ĩso(H) = 1.2*U*eq(C) or 1.5*U*eq(methyl). The H-atoms of the methyl groups C21 and C31 were disordered over six sites each with 0.5 site occupancy factors. A search for solvent-accessible voids in the crystal structure using PLATON (Spek, 2009) showed solvent accessible voids of 110 Å^3^. However, the refinement showed no electron density in the voids. This indicates that the crystal lost the solvent of crystallization by the time it was used for data collection, without collapse of the crystal lattice.

### Crystal data

**Table:**

C_16_H_14_N_2_O	*Z* = 6
*M**_r_* = 250.29	*F*(000) = 792
Triclinic, *P*1	*D*_x_ = 1.231 Mg m^−^^3^
Hall symbol: -P 1	Mo *K*α radiation, λ = 0.71073 Å
*a* = 7.4433 (2) Å	Cell parameters from 4372 reflections
*b* = 17.5444 (5) Å	θ = 2.3–27.3°
*c* = 18.0598 (7) Å	µ = 0.08 mm^−^^1^
α = 118.034 (2)°	*T* = 296 K
β = 100.217 (2)°	Prism, yellow
γ = 92.726 (1)°	0.25 × 0.21 × 0.15 mm
*V* = 2025.27 (11) Å^3^	

### Data collection

**Table:**

Bruker X8 APEXII CCD area-detector diffractometer	6445 reflections with *I* > 2σ(*I*)
Radiation source: fine-focus sealed tube	*R*_int_ = 0.028
graphite	θ_max_ = 28.0°, θ_min_ = 1.3°
φ and ω scans	*h* = −9→9
46425 measured reflections	*k* = −23→23
9750 independent reflections	*l* = −23→23

### Refinement

**Table:**

Refinement on *F*^2^	Secondary atom site location: difference Fourier map
Least-squares matrix: full	Hydrogen site location: inferred from neighbouring sites
*R*[*F*^2^ > 2σ(*F*^2^)] = 0.050	H-atom parameters constrained
*wR*(*F*^2^) = 0.166	*w* = 1/[σ^2^(*F*_o_^2^) + (0.086*P*)^2^ + 0.196*P*] where *P* = (*F*_o_^2^ + 2*F*_c_^2^)/3
*S* = 1.09	(Δ/σ)_max_ = 0.001
9750 reflections	Δρ_max_ = 0.30 e Å^−^^3^
516 parameters	Δρ_min_ = −0.24 e Å^−^^3^
0 restraints	Extinction correction: *SHELXL97* (Sheldrick, 2008), Fc^*^=kFc[1+0.001xFc^2^λ^3^/sin(2θ)]^-1/4^
Primary atom site location: structure-invariant direct methods	Extinction coefficient: 0.0084 (16)

### Special details

**Table:**

Experimental. The data collection nominally covered a sphere of reciprocal space, by a combination of four sets of exposures; each set had a different φ angle for the crystal and each exposure covered 0.5° in ω and 30 s in time. The crystal-to-detector distance was 37.5 mm.
Geometry. All e.s.d.'s (except the e.s.d. in the dihedral angle between two l.s. planes) are estimated using the full covariance matrix. The cell e.s.d.'s are taken into account individually in the estimation of e.s.d.'s in distances, angles and torsion angles; correlations between e.s.d.'s in cell parameters are only used when they are defined by crystal symmetry. An approximate (isotropic) treatment of cell e.s.d.'s is used for estimating e.s.d.'s involving l.s. planes.
Refinement. Refinement of *F*^2^ against ALL reflections. The weighted *R*-factor *wR* and goodness of fit *S* are based on *F*^2^, conventional *R*-factors *R* are based on *F*, with *F* set to zero for negative *F*^2^. The threshold expression of *F*^2^ > σ(*F*^2^) is used only for calculating *R*-factors(gt) *etc*. and is not relevant to the choice of reflections for refinement. *R*-factors based on *F*^2^ are statistically about twice as large as those based on *F*, and *R*- factors based on ALL data will be even larger.

### Fractional atomic coordinates and isotropic or equivalent isotropic displacement parameters (Å^2^)

**Table:**

	*x*	*y*	*z*	*U*_iso_*/*U*_eq_	Occ. (<1)
N11	0.28817 (17)	0.61727 (8)	0.12094 (8)	0.0539 (3)	
N12	0.24494 (17)	0.50211 (9)	−0.05373 (8)	0.0535 (3)	
N21	0.68069 (17)	0.40186 (8)	0.50730 (7)	0.0473 (3)	
N22	0.82004 (17)	0.56626 (8)	0.53631 (8)	0.0535 (3)	
N31	0.29515 (17)	0.08130 (8)	0.97102 (8)	0.0497 (3)	
N32	0.22062 (16)	−0.00511 (8)	1.06097 (8)	0.0496 (3)	
C310	0.28440 (18)	−0.00937 (9)	0.93170 (9)	0.0452 (3)	
C316	0.22919 (19)	0.07835 (10)	1.09571 (9)	0.0485 (3)	
C317	0.2642 (2)	0.12871 (10)	1.05228 (10)	0.0518 (4)	
C314	0.2368 (2)	−0.14167 (10)	0.94073 (10)	0.0557 (4)	
H314	0.2116	−0.1693	0.9716	0.067*	
C311	0.3100 (2)	−0.05966 (11)	0.84834 (10)	0.0571 (4)	
H311	0.3340	−0.0330	0.8164	0.069*	
C313	0.2644 (2)	−0.19009 (11)	0.85939 (11)	0.0635 (4)	
H313	0.2595	−0.2503	0.8352	0.076*	
C312	0.2996 (2)	−0.14864 (12)	0.81358 (11)	0.0643 (5)	
H312	0.3167	−0.1817	0.7580	0.077*	
C320	0.1937 (2)	0.12140 (10)	0.85582 (10)	0.0562 (4)	
C319	0.3461 (2)	0.12854 (12)	0.92698 (12)	0.0637 (4)	
H31A	0.4511	0.1063	0.9030	0.076*	
H31B	0.3847	0.1898	0.9692	0.076*	
C31	0.2052 (3)	0.12919 (12)	1.18591 (11)	0.0657 (4)	
H31C	0.1831	0.0899	1.2076	0.099*	0.50
H31D	0.1020	0.1601	1.1865	0.099*	0.50
H31E	0.3150	0.1702	1.2215	0.099*	0.50
H31F	0.2169	0.1902	1.2028	0.099*	0.50
H31G	0.2981	0.1200	1.2239	0.099*	0.50
H31H	0.0851	0.1100	1.1889	0.099*	0.50
C325	0.0106 (3)	0.09773 (12)	0.84936 (12)	0.0684 (5)	
H325	−0.0229	0.0836	0.8892	0.082*	
C323	−0.0772 (4)	0.11456 (15)	0.72508 (15)	0.0924 (7)	
H323	−0.1680	0.1115	0.6807	0.111*	
C321	0.2399 (3)	0.14226 (12)	0.79587 (13)	0.0766 (5)	
H321	0.3630	0.1586	0.7992	0.092*	
C322	0.1044 (4)	0.13898 (14)	0.73145 (15)	0.0938 (7)	
H322	0.1368	0.1535	0.6917	0.113*	
C324	−0.1241 (3)	0.09471 (15)	0.78441 (14)	0.0856 (6)	
H324	−0.2476	0.0790	0.7810	0.103*	
C210	0.64496 (18)	0.42088 (9)	0.43991 (8)	0.0420 (3)	
C215	0.71574 (19)	0.50356 (9)	0.45595 (9)	0.0450 (3)	
C217	0.7903 (2)	0.46041 (11)	0.58679 (9)	0.0532 (4)	
C211	0.54277 (19)	0.36064 (10)	0.35746 (9)	0.0510 (3)	
H211	0.4967	0.3050	0.3458	0.061*	
C216	0.85465 (19)	0.54651 (10)	0.59690 (9)	0.0521 (4)	
C212	0.5101 (2)	0.38320 (12)	0.29369 (10)	0.0599 (4)	
H212	0.4423	0.3426	0.2390	0.072*	
C214	0.6798 (2)	0.52521 (11)	0.39025 (11)	0.0573 (4)	
H214	0.7258	0.5805	0.4008	0.069*	
C213	0.5769 (2)	0.46550 (13)	0.30999 (11)	0.0629 (4)	
H213	0.5523	0.4806	0.2667	0.075*	
C219	0.6009 (3)	0.31832 (11)	0.49568 (11)	0.0613 (4)	
H21A	0.4733	0.3045	0.4643	0.074*	
H21B	0.6016	0.3240	0.5518	0.074*	
C21	0.9649 (2)	0.61360 (13)	0.68370 (11)	0.0733 (5)	
H21C	0.9990	0.6665	0.6826	0.110*	0.50
H21D	1.0744	0.5922	0.6985	0.110*	0.50
H21E	0.8923	0.6252	0.7258	0.110*	0.50
H21F	0.9781	0.5895	0.7220	0.110*	0.50
H21G	0.9027	0.6637	0.7061	0.110*	0.50
H21H	1.0848	0.6307	0.6788	0.110*	0.50
C220	0.7010 (3)	0.24364 (10)	0.44807 (10)	0.0605 (4)	
C225	0.8727 (3)	0.25573 (12)	0.43371 (10)	0.0639 (4)	
H225	0.9296	0.3119	0.4519	0.077*	
C224	0.9621 (3)	0.18488 (14)	0.39230 (12)	0.0816 (6)	
H224	1.0781	0.1938	0.3828	0.098*	
C222	0.7101 (6)	0.08964 (16)	0.37896 (19)	0.1197 (10)	
H222	0.6543	0.0332	0.3606	0.144*	
C223	0.8800 (5)	0.10206 (16)	0.36553 (15)	0.1040 (9)	
H223	0.9402	0.0546	0.3383	0.125*	
C221	0.6180 (4)	0.15941 (14)	0.41957 (16)	0.0984 (7)	
H221	0.5009	0.1497	0.4277	0.118*	
C115	0.21989 (19)	0.47190 (9)	0.00291 (9)	0.0480 (3)	
C110	0.24117 (18)	0.52774 (10)	0.09044 (9)	0.0474 (3)	
C116	0.2905 (2)	0.58474 (11)	−0.02376 (11)	0.0555 (4)	
C117	0.3162 (2)	0.64957 (10)	0.06708 (11)	0.0570 (4)	
C114	0.1692 (2)	0.38238 (11)	−0.03048 (12)	0.0614 (4)	
H114	0.1528	0.3451	−0.0893	0.074*	
C120	0.4972 (2)	0.69464 (11)	0.26728 (10)	0.0568 (4)	
C111	0.2174 (2)	0.49301 (13)	0.14423 (11)	0.0643 (4)	
H111	0.2334	0.5295	0.2032	0.077*	
C112	0.1700 (3)	0.40430 (15)	0.10900 (15)	0.0759 (6)	
H112	0.1554	0.3812	0.1449	0.091*	
C125	0.6317 (2)	0.64524 (12)	0.23666 (12)	0.0652 (4)	
H125	0.6074	0.6011	0.1796	0.078*	
C119	0.3077 (3)	0.68063 (12)	0.21185 (11)	0.0708 (5)	
H11A	0.2167	0.6609	0.2340	0.085*	
H11B	0.2811	0.7361	0.2165	0.085*	
C113	0.1435 (3)	0.34875 (13)	0.02172 (16)	0.0758 (5)	
H113	0.1085	0.2890	−0.0012	0.091*	
C11	0.3186 (3)	0.61802 (15)	−0.08384 (15)	0.0822 (6)	
H11C	0.4464	0.6413	−0.0712	0.123*	
H11D	0.2444	0.6632	−0.0769	0.123*	
H11E	0.2831	0.5710	−0.1421	0.123*	
C124	0.8032 (3)	0.66059 (15)	0.29001 (15)	0.0790 (6)	
H124	0.8923	0.6260	0.2686	0.095*	
C122	0.7113 (4)	0.77463 (18)	0.40426 (13)	0.0971 (8)	
H122	0.7379	0.8190	0.4613	0.117*	
C123	0.8427 (3)	0.72538 (18)	0.37299 (16)	0.0909 (7)	
H123	0.9589	0.7360	0.4082	0.109*	
C121	0.5376 (3)	0.75980 (13)	0.35231 (11)	0.0774 (5)	
H121	0.4485	0.7937	0.3747	0.093*	
C315	0.24616 (18)	−0.05134 (9)	0.97775 (9)	0.0453 (3)	
O3	0.2684 (2)	0.20807 (7)	1.08693 (8)	0.0760 (4)	
O2	0.8293 (2)	0.44281 (9)	0.64486 (8)	0.0787 (4)	
O1	0.3599 (2)	0.72780 (8)	0.09362 (10)	0.0879 (4)	

### Atomic displacement parameters (Å^2^)

**Table:**

	*U*^11^	*U*^22^	*U*^33^	*U*^12^	*U*^13^	*U*^23^
N11	0.0469 (7)	0.0550 (7)	0.0483 (7)	0.0114 (5)	0.0066 (5)	0.0171 (6)
N12	0.0517 (7)	0.0603 (8)	0.0489 (7)	0.0140 (6)	0.0127 (5)	0.0259 (6)
N21	0.0511 (7)	0.0470 (6)	0.0435 (6)	0.0113 (5)	0.0137 (5)	0.0205 (5)
N22	0.0476 (7)	0.0494 (7)	0.0524 (7)	0.0056 (5)	0.0138 (5)	0.0151 (6)
N31	0.0479 (7)	0.0470 (7)	0.0509 (7)	−0.0003 (5)	0.0031 (5)	0.0246 (6)
N32	0.0458 (6)	0.0501 (7)	0.0479 (7)	0.0031 (5)	0.0041 (5)	0.0224 (6)
C310	0.0336 (6)	0.0473 (7)	0.0459 (7)	0.0031 (5)	0.0010 (5)	0.0186 (6)
C316	0.0390 (7)	0.0520 (8)	0.0447 (7)	0.0048 (6)	0.0010 (6)	0.0189 (6)
C317	0.0483 (8)	0.0447 (8)	0.0525 (8)	0.0058 (6)	−0.0002 (6)	0.0198 (7)
C314	0.0514 (8)	0.0469 (8)	0.0596 (9)	0.0047 (6)	0.0000 (7)	0.0229 (7)
C311	0.0469 (8)	0.0693 (10)	0.0509 (9)	0.0095 (7)	0.0082 (6)	0.0268 (8)
C313	0.0579 (9)	0.0472 (8)	0.0626 (10)	0.0095 (7)	−0.0029 (8)	0.0140 (8)
C312	0.0512 (9)	0.0676 (11)	0.0486 (9)	0.0160 (8)	0.0039 (7)	0.0097 (8)
C320	0.0682 (10)	0.0431 (8)	0.0592 (9)	0.0066 (7)	0.0138 (8)	0.0269 (7)
C319	0.0604 (10)	0.0614 (10)	0.0710 (11)	−0.0056 (8)	0.0077 (8)	0.0374 (9)
C31	0.0625 (10)	0.0682 (11)	0.0505 (9)	0.0096 (8)	0.0087 (7)	0.0176 (8)
C325	0.0663 (11)	0.0806 (12)	0.0669 (11)	0.0178 (9)	0.0156 (8)	0.0421 (10)
C323	0.124 (2)	0.0773 (14)	0.0761 (14)	0.0410 (14)	0.0033 (13)	0.0420 (11)
C321	0.1011 (15)	0.0621 (11)	0.0762 (12)	−0.0015 (10)	0.0203 (11)	0.0425 (10)
C322	0.149 (2)	0.0725 (13)	0.0768 (14)	0.0162 (14)	0.0190 (14)	0.0522 (12)
C324	0.0730 (13)	0.0944 (15)	0.0890 (15)	0.0303 (11)	0.0106 (11)	0.0454 (13)
C210	0.0379 (6)	0.0459 (7)	0.0407 (7)	0.0132 (5)	0.0134 (5)	0.0177 (6)
C215	0.0408 (7)	0.0458 (7)	0.0452 (7)	0.0125 (6)	0.0148 (6)	0.0173 (6)
C217	0.0488 (8)	0.0673 (10)	0.0414 (8)	0.0208 (7)	0.0129 (6)	0.0227 (7)
C211	0.0434 (7)	0.0507 (8)	0.0458 (8)	0.0066 (6)	0.0073 (6)	0.0140 (7)
C216	0.0410 (7)	0.0567 (9)	0.0443 (8)	0.0106 (6)	0.0125 (6)	0.0118 (7)
C212	0.0471 (8)	0.0801 (12)	0.0420 (8)	0.0194 (8)	0.0070 (6)	0.0214 (8)
C214	0.0626 (9)	0.0594 (9)	0.0636 (10)	0.0213 (8)	0.0262 (8)	0.0357 (8)
C213	0.0635 (10)	0.0858 (12)	0.0538 (9)	0.0310 (9)	0.0205 (8)	0.0413 (9)
C219	0.0725 (11)	0.0584 (9)	0.0606 (10)	0.0103 (8)	0.0242 (8)	0.0318 (8)
C21	0.0546 (10)	0.0806 (12)	0.0495 (9)	0.0060 (8)	0.0045 (7)	0.0066 (8)
C220	0.0866 (12)	0.0531 (9)	0.0464 (8)	0.0136 (8)	0.0136 (8)	0.0281 (7)
C225	0.0734 (11)	0.0629 (10)	0.0484 (9)	0.0207 (8)	0.0055 (8)	0.0233 (8)
C224	0.0912 (14)	0.0869 (15)	0.0557 (10)	0.0400 (12)	0.0099 (9)	0.0259 (10)
C222	0.195 (3)	0.0580 (13)	0.112 (2)	0.0256 (17)	0.057 (2)	0.0380 (13)
C223	0.166 (3)	0.0746 (15)	0.0710 (14)	0.0575 (17)	0.0278 (16)	0.0313 (12)
C221	0.142 (2)	0.0606 (12)	0.1010 (17)	0.0127 (13)	0.0490 (16)	0.0386 (12)
C115	0.0429 (7)	0.0512 (8)	0.0516 (8)	0.0139 (6)	0.0087 (6)	0.0265 (7)
C110	0.0382 (7)	0.0557 (8)	0.0485 (8)	0.0123 (6)	0.0062 (6)	0.0262 (7)
C116	0.0470 (8)	0.0649 (10)	0.0633 (10)	0.0120 (7)	0.0133 (7)	0.0375 (8)
C117	0.0477 (8)	0.0505 (9)	0.0682 (10)	0.0075 (7)	0.0083 (7)	0.0268 (8)
C114	0.0579 (9)	0.0512 (9)	0.0678 (10)	0.0137 (7)	0.0070 (8)	0.0250 (8)
C120	0.0617 (9)	0.0572 (9)	0.0477 (8)	0.0030 (7)	0.0102 (7)	0.0237 (7)
C111	0.0510 (9)	0.0926 (13)	0.0583 (10)	0.0156 (8)	0.0073 (7)	0.0451 (10)
C112	0.0582 (10)	0.0997 (15)	0.1035 (16)	0.0136 (10)	0.0114 (10)	0.0784 (14)
C125	0.0562 (9)	0.0684 (11)	0.0665 (10)	0.0052 (8)	0.0084 (8)	0.0316 (9)
C119	0.0623 (10)	0.0692 (11)	0.0550 (10)	0.0195 (8)	0.0124 (8)	0.0091 (8)
C113	0.0636 (11)	0.0625 (11)	0.1107 (17)	0.0117 (8)	0.0063 (10)	0.0540 (12)
C11	0.0773 (13)	0.1012 (15)	0.0964 (15)	0.0114 (11)	0.0272 (11)	0.0683 (13)
C124	0.0596 (11)	0.0971 (15)	0.0982 (16)	0.0023 (10)	0.0034 (10)	0.0674 (13)
C122	0.1140 (19)	0.1139 (19)	0.0466 (10)	−0.0243 (15)	−0.0085 (11)	0.0383 (11)
C123	0.0792 (14)	0.128 (2)	0.0820 (15)	−0.0175 (14)	−0.0125 (12)	0.0770 (16)
C121	0.0951 (14)	0.0802 (13)	0.0491 (10)	0.0050 (10)	0.0163 (9)	0.0259 (9)
C315	0.0355 (6)	0.0461 (7)	0.0466 (8)	0.0026 (5)	−0.0001 (5)	0.0199 (6)
O3	0.0989 (10)	0.0443 (6)	0.0733 (8)	0.0121 (6)	0.0078 (7)	0.0233 (6)
O2	0.0898 (9)	0.0995 (10)	0.0531 (7)	0.0288 (7)	0.0113 (6)	0.0425 (7)
O1	0.0865 (10)	0.0527 (7)	0.1109 (11)	−0.0007 (6)	0.0136 (8)	0.0328 (7)

### Geometric parameters (Å, °)

**Table:**

N11—C117	1.375 (2)	C212—C213	1.378 (3)
N11—C110	1.3996 (19)	C212—H212	0.9300
N11—C119	1.462 (2)	C214—C213	1.374 (2)
N12—C116	1.288 (2)	C214—H214	0.9300
N12—C115	1.3891 (19)	C213—H213	0.9300
N21—C217	1.3784 (19)	C219—C220	1.507 (2)
N21—C210	1.3925 (18)	C219—H21A	0.9700
N21—C219	1.459 (2)	C219—H21B	0.9700
N22—C216	1.284 (2)	C21—H21C	0.9600
N22—C215	1.3920 (19)	C21—H21D	0.9600
N31—C317	1.374 (2)	C21—H21E	0.9600
N31—C310	1.3954 (18)	C21—H21F	0.9600
N31—C319	1.470 (2)	C21—H21G	0.9600
N32—C316	1.2866 (19)	C21—H21H	0.9600
N32—C315	1.3868 (18)	C220—C225	1.375 (3)
C310—C311	1.397 (2)	C220—C221	1.384 (3)
C310—C315	1.398 (2)	C225—C224	1.388 (2)
C316—C317	1.472 (2)	C225—H225	0.9300
C316—C31	1.495 (2)	C224—C223	1.367 (4)
C317—O3	1.2251 (18)	C224—H224	0.9300
C314—C313	1.369 (2)	C222—C223	1.355 (4)
C314—C315	1.393 (2)	C222—C221	1.386 (4)
C314—H314	0.9300	C222—H222	0.9300
C311—C312	1.374 (2)	C223—H223	0.9300
C311—H311	0.9300	C221—H221	0.9300
C313—C312	1.381 (3)	C115—C110	1.387 (2)
C313—H313	0.9300	C115—C114	1.395 (2)
C312—H312	0.9300	C110—C111	1.398 (2)
C320—C325	1.374 (3)	C116—C117	1.462 (2)
C320—C321	1.384 (2)	C116—C11	1.492 (2)
C320—C319	1.507 (2)	C117—O1	1.2261 (19)
C319—H31A	0.9700	C114—C113	1.360 (3)
C319—H31B	0.9700	C114—H114	0.9300
C31—H31C	0.9600	C120—C125	1.374 (2)
C31—H31D	0.9600	C120—C121	1.382 (2)
C31—H31E	0.9600	C120—C119	1.512 (2)
C31—H31F	0.9600	C111—C112	1.374 (3)
C31—H31G	0.9600	C111—H111	0.9300
C31—H31H	0.9600	C112—C113	1.379 (3)
C325—C324	1.379 (3)	C112—H112	0.9300
C325—H325	0.9300	C125—C124	1.385 (3)
C323—C324	1.365 (3)	C125—H125	0.9300
C323—C322	1.367 (4)	C119—H11A	0.9700
C323—H323	0.9300	C119—H11B	0.9700
C321—C322	1.374 (3)	C113—H113	0.9300
C321—H321	0.9300	C11—H11C	0.9600
C322—H322	0.9300	C11—H11D	0.9600
C324—H324	0.9300	C11—H11E	0.9600
C210—C215	1.394 (2)	C124—C123	1.355 (3)
C210—C211	1.3983 (19)	C124—H124	0.9300
C215—C214	1.394 (2)	C122—C123	1.357 (4)
C217—O2	1.2185 (19)	C122—C121	1.388 (3)
C217—C216	1.478 (2)	C122—H122	0.9300
C211—C212	1.371 (2)	C123—H123	0.9300
C211—H211	0.9300	C121—H121	0.9300
C216—C21	1.495 (2)		
			
C117—N11—C110	121.79 (13)	N21—C219—C220	113.86 (14)
C117—N11—C119	117.13 (14)	N21—C219—H21A	108.8
C110—N11—C119	121.08 (14)	C220—C219—H21A	108.8
C116—N12—C115	118.81 (13)	N21—C219—H21B	108.8
C217—N21—C210	121.92 (13)	C220—C219—H21B	108.8
C217—N21—C219	117.66 (13)	H21A—C219—H21B	107.7
C210—N21—C219	120.41 (12)	C216—C21—H21C	109.5
C216—N22—C215	118.75 (13)	C216—C21—H21D	109.5
C317—N31—C310	121.23 (12)	H21C—C21—H21D	109.5
C317—N31—C319	118.47 (13)	C216—C21—H21E	109.5
C310—N31—C319	120.27 (13)	H21C—C21—H21E	109.5
C316—N32—C315	118.81 (13)	H21D—C21—H21E	109.5
N31—C310—C311	122.63 (14)	C216—C21—H21F	109.5
N31—C310—C315	118.51 (13)	H21C—C21—H21F	141.1
C311—C310—C315	118.85 (14)	H21D—C21—H21F	56.3
N32—C316—C317	123.92 (13)	H21E—C21—H21F	56.3
N32—C316—C31	119.39 (15)	C216—C21—H21G	109.5
C317—C316—C31	116.69 (14)	H21C—C21—H21G	56.3
O3—C317—N31	122.14 (15)	H21D—C21—H21G	141.1
O3—C317—C316	122.08 (15)	H21E—C21—H21G	56.3
N31—C317—C316	115.77 (13)	H21F—C21—H21G	109.5
C313—C314—C315	120.65 (16)	C216—C21—H21H	109.5
C313—C314—H314	119.7	H21C—C21—H21H	56.3
C315—C314—H314	119.7	H21D—C21—H21H	56.3
C312—C311—C310	119.83 (16)	H21E—C21—H21H	141.1
C312—C311—H311	120.1	H21F—C21—H21H	109.5
C310—C311—H311	120.1	H21G—C21—H21H	109.5
C314—C313—C312	119.32 (15)	C225—C220—C221	118.69 (18)
C314—C313—H313	120.3	C225—C220—C219	122.86 (16)
C312—C313—H313	120.3	C221—C220—C219	118.43 (19)
C311—C312—C313	121.41 (16)	C220—C225—C224	120.62 (19)
C311—C312—H312	119.3	C220—C225—H225	119.7
C313—C312—H312	119.3	C224—C225—H225	119.7
C325—C320—C321	118.56 (17)	C223—C224—C225	120.2 (2)
C325—C320—C319	122.91 (15)	C223—C224—H224	119.9
C321—C320—C319	118.50 (16)	C225—C224—H224	119.9
N31—C319—C320	114.42 (13)	C223—C222—C221	121.2 (3)
N31—C319—H31A	108.7	C223—C222—H222	119.4
C320—C319—H31A	108.7	C221—C222—H222	119.4
N31—C319—H31B	108.7	C222—C223—C224	119.5 (2)
C320—C319—H31B	108.7	C222—C223—H223	120.3
H31A—C319—H31B	107.6	C224—C223—H223	120.3
C316—C31—H31C	109.5	C220—C221—C222	119.8 (3)
C316—C31—H31D	109.5	C220—C221—H221	120.1
H31C—C31—H31D	109.5	C222—C221—H221	120.1
C316—C31—H31E	109.5	C110—C115—N12	122.21 (13)
H31C—C31—H31E	109.5	C110—C115—C114	119.57 (14)
H31D—C31—H31E	109.5	N12—C115—C114	118.22 (14)
C316—C31—H31F	109.5	C115—C110—C111	119.38 (15)
H31C—C31—H31F	141.1	C115—C110—N11	117.70 (13)
H31D—C31—H31F	56.3	C111—C110—N11	122.91 (14)
H31E—C31—H31F	56.3	N12—C116—C117	123.73 (14)
C316—C31—H31G	109.5	N12—C116—C11	119.35 (16)
H31C—C31—H31G	56.3	C117—C116—C11	116.91 (16)
H31D—C31—H31G	141.1	O1—C117—N11	122.09 (16)
H31E—C31—H31G	56.3	O1—C117—C116	122.16 (17)
H31F—C31—H31G	109.5	N11—C117—C116	115.74 (13)
C316—C31—H31H	109.5	C113—C114—C115	121.00 (17)
H31C—C31—H31H	56.3	C113—C114—H114	119.5
H31D—C31—H31H	56.3	C115—C114—H114	119.5
H31E—C31—H31H	141.1	C125—C120—C121	118.34 (17)
H31F—C31—H31H	109.5	C125—C120—C119	122.77 (14)
H31G—C31—H31H	109.5	C121—C120—C119	118.90 (16)
C320—C325—C324	120.63 (19)	C112—C111—C110	119.25 (17)
C320—C325—H325	119.7	C112—C111—H111	120.4
C324—C325—H325	119.7	C110—C111—H111	120.4
C324—C323—C322	119.4 (2)	C111—C112—C113	121.61 (17)
C324—C323—H323	120.3	C111—C112—H112	119.2
C322—C323—H323	120.3	C113—C112—H112	119.2
C322—C321—C320	120.3 (2)	C120—C125—C124	120.60 (19)
C322—C321—H321	119.9	C120—C125—H125	119.7
C320—C321—H321	119.9	C124—C125—H125	119.7
C323—C322—C321	120.7 (2)	N11—C119—C120	113.91 (14)
C323—C322—H322	119.6	N11—C119—H11A	108.8
C321—C322—H322	119.6	C120—C119—H11A	108.8
C323—C324—C325	120.4 (2)	N11—C119—H11B	108.8
C323—C324—H324	119.8	C120—C119—H11B	108.8
C325—C324—H324	119.8	H11A—C119—H11B	107.7
N21—C210—C215	118.06 (12)	C114—C113—C112	119.15 (17)
N21—C210—C211	122.81 (13)	C114—C113—H113	120.4
C215—C210—C211	119.13 (13)	C112—C113—H113	120.4
N22—C215—C210	122.03 (13)	C116—C11—H11C	109.5
N22—C215—C214	118.47 (14)	C116—C11—H11D	109.5
C210—C215—C214	119.49 (14)	H11C—C11—H11D	109.5
O2—C217—N21	122.38 (16)	C116—C11—H11E	109.5
O2—C217—C216	122.44 (15)	H11C—C11—H11E	109.5
N21—C217—C216	115.18 (13)	H11D—C11—H11E	109.5
C212—C211—C210	120.35 (15)	C123—C124—C125	120.7 (2)
C212—C211—H211	119.8	C123—C124—H124	119.6
C210—C211—H211	119.8	C125—C124—H124	119.6
N22—C216—C217	123.92 (13)	C123—C122—C121	120.9 (2)
N22—C216—C21	119.22 (16)	C123—C122—H122	119.5
C217—C216—C21	116.85 (15)	C121—C122—H122	119.5
C211—C212—C213	120.53 (15)	C124—C123—C122	119.4 (2)
C211—C212—H212	119.7	C124—C123—H123	120.3
C213—C212—H212	119.7	C122—C123—H123	120.3
C213—C214—C215	120.48 (16)	C120—C121—C122	120.0 (2)
C213—C214—H214	119.8	C120—C121—H121	120.0
C215—C214—H214	119.8	C122—C121—H121	120.0
C214—C213—C212	120.00 (15)	N32—C315—C314	118.37 (14)
C214—C213—H213	120.0	N32—C315—C310	121.69 (13)
C212—C213—H213	120.0	C314—C315—C310	119.92 (14)

### Hydrogen-bond geometry (Å, °)

**Table:**

*D*—H···*A*	*D*—H	H···*A*	*D*···*A*	*D*—H···*A*
C119—H11B···O1	0.97	2.34	2.706 (3)	102
C219—H21B···O2	0.97	2.32	2.722 (2)	104
C21—H21F···O2	0.96	2.37	2.824 (3)	109
C319—H31B···O3	0.97	2.33	2.740 (2)	105
C31—H31F···O3	0.96	2.35	2.810 (3)	109
C125—H125···N11	0.93	2.51	2.858 (2)	102
C125—H125···N12^i^	0.93	2.62	3.423 (2)	145
C225—H225···N21	0.93	2.53	2.864 (3)	102
C321—H321···O1^ii^	0.93	2.56	3.320 (3)	140
C325—H325···N31	0.93	2.57	2.898 (3)	101
C325—H325···N32^iii^	0.93	2.51	3.380 (3)	155

Symmetry codes: (i) −*x*+1, −*y*+1, −*z*; (ii) −*x*+1, −*y*+1, −*z*+1; (iii) −*x*, −*y*, −*z*+2.
